# Acne-induced Post-inflammatory Hyperpigmentation: From Grading to Treatment

**DOI:** 10.2340/actadv.v105.42925

**Published:** 2025-04-22

**Authors:** Nicole AUFFRET, Marie-Thérèse LECCIA, Fabienne BALLANGER, Jean Paul CLAUDEL, Serge DAHAN, Brigitte DRÉNO

**Affiliations:** 1 Private practice, Paris; 2 Department of Dermatology, Allergology and Photobiology, CHU A Michallon, Grenoble; 3 Private practice, Talencee; 4 Private practice, Tours; 5 Dermatology, Aesthetic and Lasers, Toulouse; 6 Nantes Université, INSERM, CNRS, Immunology and New Concepts in ImmunoTherapy, INCIT, UMR 1302/EMR6001, Nantes, France

**Keywords:** acne, post-inflammatory hyperpigmentation, treatments, retinoids

## Abstract

Acne-induced post-inflammatory hyperpigmentation (AI-PIH) can occur without any visible clinical evidence of significant inflammation, even in patients with mild to moderate acne. Currently, visual assessment is the main criterion for evaluating the severity of PIH, including that of AI-PIH in daily clinical practice. Treatment indications are lacking. This work provides an easy-to-use AI-PIH severity grading tool for daily clinical practice as well as indications on how to prevent and treat AI-PIH using currently available treatment options. Five experts in acne provided a short overview concerning the epidemiology and physiopathology of AI-PIH, developed an AI-PIH severity grading tool, and proposed preventive measures as well as an AI-PIH treatment algorithm. Only a small number of epidemiological data on AI-PIH are available, confirming that the condition is mainly observed in patients with Phototypes IV to VI. The physiopathology of AI-PIH is still not completely understood. Innate immunity, *Cutibacterium acnes,* and external factors such as UV radiation, visible light, and air pollution play a role in its development. An easy-to-use AI-PIH severity grading tool (Acne PIgmentation Grading) allows quick assessment of acne severity during the consultation, and, in addition to proposed preventive measures, a treatment algorithm is proposed according to AI-PH severity. Patient education remains key. Providing an AI-PIH severity assessment tool as well as preventive and treatment recommendations may help to manage AI-PIH more efficiently.

## SIGNIFICANCE

Acne-induced post-inflammatory hyperpigmentation can occur without any visible clinical evidence of significant inflammation, even in patients with mild to moderate acne. In daily clinical practice, visual assessment is the main criterion for evaluating the severity of acne-induced postinflammatory hyperpigmentation. However, easy-to-use scoring tools and an easy-to-follow treatment algorithm to be used in daily clinical practice are lacking. This article provides for the first time an easy-to-use scoring tool and an easy-to-follow treatment algorithm developed for acneinduced post-inflammatory hyperpigmentation.

*Acne vulgaris*, or acne, is a common chronic inflammatory skin disease. It is commonly observed in adolescents and is more and more frequently described in adults (1). Acne affects all skin phototypes, causing scars in its more severe forms, as well as post-inflammatory hyperpigmentation (PIH) marks (2). PIH is a widespread, acquired pigmentary disorder caused by cutaneous endogenous inflammation such as acne, external injury, or cutaneous procedures (3). In subjects with skin of colour, inflammation may be subclinical or difficult to observe (4).

PIH is more frequently observed among patients with Fitzpatrick skin phototypes III to VI than in those with skin types I to III (5). This is due to increased size and quantity of melanosomes (6). PIH heavily impacts patients’ quality of life (QoL) and prevention, along with suitable early management, helps to reduce the risk of PIH and to improve the patient’s QoL (7). Despite its prevalence, AI-PIH is still inconsistently studied as an outcome in clinical trials, especially in patients with skin phototypes III to VI (8).

Acne induced-PIH (AI-PIH) can occur without any visible clinical evidence of significant inflammation, even in patients with mild to moderate acne (9). Currently, visual assessment is the main criterion for evaluating the severity of PIH, including that of AI-PIH in daily clinical practice (2).

Following a summary of recent epidemiological and pathophysiological data, this article provides the first AI-PIH severity grading tool that can be easily used in daily practice as well as indications on how to prevent and treat AI-PIH using currently available treatment options.

## MATERIAL AND METHODS

A group of 5 dermatologists from different institutions and private practices in France involved in the research and clinical field of acne briefly present epidemiology and pathogenesis data concerning AI-PIH published since 2000 and available from the PubMed database and propose an easy-to-use AI-PIH severity grading tool as well as preventive means and a treatment algorithm for AI-PIH.

## RESULTS

### Epidemiology

It is currently estimated that pigmentation disorders are the 11th most common condition observed by dermatologists (10). Several studies have reported that PIH, including AI-PIH, is most common in African American (20% of the diagnosed PIH) and Hispanic populations (6.0–7.5%) (11).

However, only 4 publications have provided epidemiological data on AI-PIH. An international group of dermatologists evaluated the occurrence and characteristics of AI-PIH in 324 patients with acne from several countries in Southeast Asia (12). A large majority (80.2%) of these subjects had mild to moderate acne, 63% were female and 58.2% had AI-PIH. Moreover, AI-PIH frequently lasted for at least 1 year in more than half of the subjects, and lasted 5 years or longer in 22.3% of them. Another study conducted in the Middle East with patients of phototype IV and above reported that AI-PIH was present in 87.2% of the 262 participating subjects (13). Overall, 52.6% reported that AI-PIH had been present for at least 1 year.

In a study conducted by Perkins et al. including 2,895 subjects (384 African American, 520 Asian, 1,295 Caucasian, 258 Hispanic, and 438 Continental Indian) the prevalence of AI-PIH has been reported to be 65%, 48%, and 25% for African American, Hispanic, and Caucasian patients, respectively (14). Finally, a population study reported AI-PIH in around 47–65% of African American, Hispanic, and Southeast Asian patients (15).

Considering all the above studies, shown data confirm that AI-PIH is frequently observed in patients with dark skin (phototype IV to VI).

### Physiopathology

**Fig. 1:**
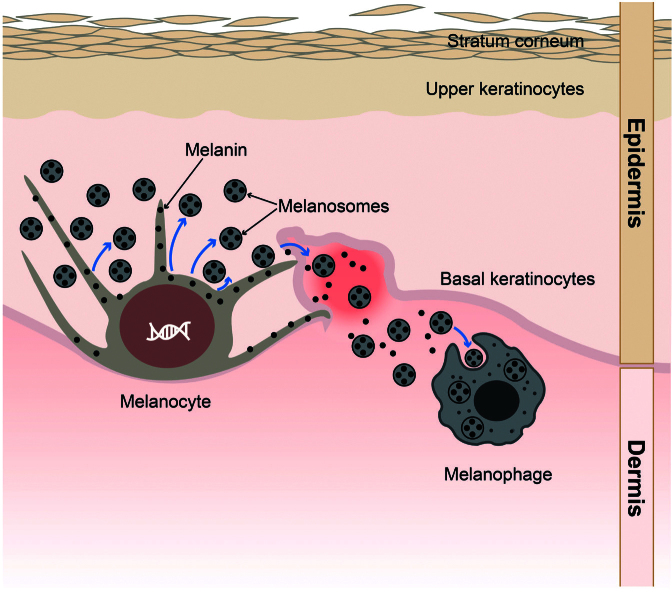
**Acne induced-post-inflammatory hyperpigmentation model (adapted from Maghfour et al. [21]).** After the release of inflammatory cytokines from keratinocytes and growth factors from dermal fibroblasts following UV exposure or trauma/injury, melanocytes release melanin in the epidermis. Epidermal ultraviolet (UV)-protective melanin may enter the dermis via a damaged basal cell layer where it is phagocytized creating melanophages.

The exact signalling pathway of AI-PIH has still not been completely elucidated (16). In AI-PIH, inflammation stimulates melanogenesis and pigment deposition (**Fig. 1**) (2).

UV radiation, especially UVA, increases the thickness of the stratum corneum and alters the skin microbiome (17, 18). As a result, the number of closed comedones may increase, leading to inflammation and acne flare-ups, especially in skin phototypes IV to VI, and in patients with severe inflammatory acne according to the GEA grading (19, 20).

Recent data confirm that AI-PIH involves mainly the epidermis and sometimes the dermis (21).

Increasing evidence has identified the role of inflammation at all stages of acne, even at the subclinical level prior to the formation of comedones, with a lymphocytic perivascular infiltrate as early as 6 h after the onset of an acne lesion, including microcomedones (22, 23). In healthy skin, inflammation was observed before any hyperproliferative changes (24).

The release of arachidonic acid in response to epidermal inflammation has been considered a cause of oxidation of prostaglandins, leukotrienes, and other molecules. This may stimulate the melanocyte activity dynamics as well as the innate immune response to *Cutibacterium acnes* (2, 21, 25, 26). *In vitro*, these metabolites increase the melanocyte size and trigger the proliferation of melanocyte dendritic cells. Moreover, leukotriene C4 increases the tyrosinase activity while the release of cytokines and inflammatory mediators including interleukins 1a and 6, tumour necrosis factor a, endothelin-1, stem cell factor, basic fibroblast growth factor, superoxide, and nitric oxide stimulate melanin production (2). The increase of melanin production results in increased melanin distribution to close keratinocytes (27). In addition, epidermal ultraviolet (UV)-protective melanin enters the dermis via a damaged basal cell layer. There it is phagocytized by macrophages, creating melanophages. It is thought that the intensity of the pigmentary response correlates with the degree and long-term and/or relapsing of inflammation (28).

In acne, innate immunity stimulated by *Cutibacterium acnes* plays a major role in the physiopathology via toll-like receptor (TLR) 2 (29).

A recent hypothesis suggests that melanocytes express functional TLRs. TLRs stimulate innate immune stimulation affecting melanin synthesis and melanosome transport to modulate skin pigmentation (30). TLR2 enhances melanogenetic gene expression to augment melanogenesis. TLR4 and TLR9 enhance tyrosinase expression and melanogenesis through p38 MAPK (mitogen-activated protein kinase) and the NF_k_B signalling pathway, respectively. TLR7 suppresses microphthalmia-associated transcription factor (MITF), and MITF reduction leads to melanocyte apoptosis. Increased knowledge regarding the role of TLR in the innate immunity in acne and of melanogenesis may help to elucidate the interplay between acne and melanogenesis. Therefore, a balanced skin microbiome is not only important to maintain the natural skin barrier and to improve acne, but also may help to limit or avoid AI-PIH.

### Clinical picture of AI-PIH

AI-Pih is the most frequently observed in Phototypes IV to VI. AI-PIH most frequently occurs in patients with Phototypes IV to VI with moderate or severe forms of acne (31, 32). However, it may also be observed in milder forms of acne. AI-PIH manifests as localized or diffuse brown-to-grey brown macules at the sites of acne lesions on cheeks and the mandibular area (2, 33). It becomes most apparent after lesional erythema has resolved (34).

AI-PIH may also be violaceous, which may be a sign of resolving acne. In this case, inflammation contributes to a violaceous discoloration, rather than brownish pigment. In dark skin, erythema may not be easily visible, and the only clinical sign of inflammation may be a more subtle violaceous shade. This is of special importance when making management decisions (16).

### Assessment

The clinical and instrumental assessment of AI-PIH may be challenging, and assessing AI-PIH has been considered a major issue, particularly in Asian populations (16, 34). Goh et al. evaluated the concordance in the diagnoses of PIH in Asian patients with acne, acne scarring, and pigmentation disorders. The study showed that there was a significant variability in rating the presence of PIH, with an average of 30 diagnoses (24%) difference between high- and low-frequency raters. Most cases were considered as mild, with a marked variability between raters. The variability was greatest when subjects also had acne (34).

Common, non-invasive instrumental equipment exists to detect and quantify PIH, and may be used in daily clinical practice to assess AI-PIH. These devices include Wood’s Lamp, UV light photography, parallel-polarized light photography, cross polarized photography, colorimetry and diffuse reflectance spectroscopy, as well as reflectance confocal microscopy (2). All these techniques are reliable and easy to use, and are currently used in clinical studies. They allow the acquisition of quantitative and objective information on PIH lesions, and for their clinical assessment to be improved. However, some of them are expensive, require a minimum of training, and may not be always useable during consultations.

The clinical assessment of the severity of PIH, including that induced by acne, remains physician-dependent (35). A bi-dimensional approach may help to efficiently and objectively assess PIH. To date, only 1 tool that assesses AI-PIH exists. This scale, PAHPI (Post-Acne Hyperpigmentation Index), was proposed by Savory et al. in 2014 (35). However, this tool is exclusively used in clinical studies and since its creation no other clinical tools that may be easily used in current clinical practice have been developed to efficiently assess the severity of AI-PIH in daily practice (36, 37).

To allow the dermatologists to assess the severity of AI-PIH in their daily clinical practice easily and quickly, we propose a 9-point grading tool (from 1 = almost no AI-PIH to 10 = severe AI-PIH) based on our experience as dermatologists.

This tool takes into consideration the phototype, duration, intensity, the number of PIH macules, and their size. We attributed specific scores to each item, considering their importance regarding AI-PIH. This tool, APIG (Acne PIgmentation Grading) is presented in **Table I**.

**Table I:** Acne induced-post-inflammatory hyperpigmentation severity grading tool APIG (Acne-induced PIgmentation Grading)

Evaluation criteria acne-induced-PIH	Score
Phototype	
I–II (0 points)	
III (1 point)	
IV–VI (2 points)	
Duration	
≤ 1 year (1 point)	
> 1 year (3 points)	
Intensity	
Slightly to moderately darker than surrounding skin (1 point)	
Significantly darker than surrounding skin (3 points)	
Number	
≤ 5 pigmentary macules (0 point)	
> 5 pigmentary macules (1 point)	
Pigmentary macule size	
≤ 6mm (0 point)	
> 6mm (1 point)	
Total dcore (2 to 10 points)	

During a first step, we defined, based on the literature review and the experience of experts, the different parameters that influence AI-PIH.

We considered the phototype as being the most important parameter, followed by the duration since first onset of AI-PIH. AI-PIH, like other PIHs, is only rarely observed in fair skin; its frequency increases with darker skin types. For that reason, we scored AI-PIH in phototype I/II with 0 points, in phototype III with 1 point, and in phototypes IV to VI with 2 points. Due to the fact that AI-PIH may resolve without treatment and when being observed for less than 1 year the impact on the patient’s QoL and burden than when observed for more than 1 year we attributed a score of 1 point for a duration of less than 1 year and 3 points for a duration of at least 1 year, considering the status of AI-PIH as being chronic.

In a second step we considered the intensity, the number of macules, and their size. A slightly to moderately greater intensity than the surrounding skin was scored with1 point and a significant intensity with *3 points.*

### Prevention and treatment

AI-PIH care is phototype dependent; it occurs more frequently in phototype IV and above. Prevention of AI-PIH is strongly recommended, especially in patients with a high risk of PIH (39). However, even subjects with lower phototypes should benefit from effective prevention (17). AI-PIH prevention consists of the daily application of emollients in the morning, as well as the use of broad-spectrum sunscreens with a sun protecting factor (SPF) of 50+, as well as mandatory UVB/UVA (> 370 nm) protection ≤ 3 covering blue light (tinted or containing iron oxides).

Sprayable formulas of organic sunscreens with a non-greasy texture and water or light liquid base show better cosmetic acceptability, and may help teenagers with acne-prone skin to adhere to its use more (17). These sunscreens frequently contain niacinamide (Vitamin B3), licochalcone, carotenoids (beta-carotene), vitamin E, vitamin C, glycyrrhetinic acid, and diethylhexyl syringylidenemalonate (DESM) (41).

In addition, patients with Phototype IV to VI may apply anti-pigmentation dermocosmetics twice daily. When staying outside for a prolonged period, subjects should also wear a sun hat (23). Prolonged exposure to airborne particulate matter (PM) and polycyclic aromatic hydrocarbons (PAHs) produces reactive oxygen species (ROS) in the skin. PM increases the amount of ROS (41). ROS triggers the increase of metalloproteinases, which increases the risk of pigmentation disorders, including AI-PIH. Therefore, prolonged exposure to polluted air should be avoided, whenever possible. **Table II** provides a short summary of preventive measures.

**Table II:** Acne induced-post-inflammatory hyperpigmentation preventive measures according to phototypes

Phototype I–II	Phototype III–VI
In the morning:	In the morning:
Emollient cream	Emollient cream
Regular use during the day:	Anti-hyperpigmentation dermocosmetics**
Sunscreen*	Regular use during the day:
	Sunscreen*

* SPF50+ broad-spectrum sunscreens (ideally tinted to protect from visible light as well) with UVB (/ UVA (> 370nm) ≤ 3 and covering blue light (tinted sunscreen, iron oxides), organic components:: i.e., niacinamide (vitamin B3), licochalcone, carotenoids (beta-carotene), vitamin E, vitamin C, glycyrrhetinic acid, diethylhexyl syringylidenemalonate (DESM).

** Dermocosmetics containing anti-pigmentation components: i.e., niacinamide (vitamin B3), liquorice (Glycyrrhiza glabra), kojic acid, alpha-hydroxy acid, botanicals, resveratrol, antioxidants (vitamins C and E).

Current treatment options include topicals, as well as one oral treatment (42, 43). **Table III** provides a list of agents currently proposed to manage AI-PIH.

**Table III:** Depigmentation agents (16)

Depigmenting agents	Mode of action
Pharmaceutical agents	
Topical retinoids	Interfering in the tyrosinase pathway and increasing epidermal turnover
Azelaic acid	Interfering in the tyrosinase pathway in inhibiting tyrosinase activity
Hydroquinone	
Tranexamic acid (oral)	Inhibiting UV plasmin-induced activity in keratinocytes
Cosmetic agents	
Antioxidants: ascorbic acid, alpha-tocopherol, 6-hydroxy-3,4-dihydrocoumarin, resveratrol, alpha-lipoic acid	Interrupting irritant-induced melanogenesis
Inhibition of tyrosinase activity: arbutin, ascorbic acid, kojic acid, 4-n-butylresorcinol, liquorice extracts	
Reduction in tyrosinase production: ceramide, sphingosine-1-phosphate	
Increase of tyrosinase degradation: hydro-quinone, linoleic acid, linolenic acid, oleic acid	
Impact on melanosomes: niacinamide, lectins, neoglycoproteins, arbutin, soy trypsin inhibitor	Inhibiting melanosome transfer/maturation

Retinoids such as tretinoin, adapalene, tazarotene, and trifarotene are not only efficacious in acne because of their anti-inflammatory properties, reducing inflammatory acne, but they also reduce acne-related sequelae such as scars and AI–PIH (39, 44–47). Retinoids in a fixed combination with benzoyl peroxide provided partial AI-PIH treatment success in patients with skin of colour (36, 48). Recently, the importance of treating acne sequelae including PIH in skin of colour was confirmed by a 6-month phase IV study of a topical 4th-generation retinoid combined with an appropriate skincare routine including UV protection, and reconfirmed in a second study (47, 50). However, AI-PIH treatment with topical retinoids may also cause iatrogenic PIH due to irritant contact dermatitis from topical acne regimens, especially retinoids (4, 21). Other topical treatments for AI-PIH include azelaic acid (AA) and hydroquinone (HQ). Orally administered tranexamic acid has recently been reported to be beneficial in hyperpigmentation disorders (50–52).

While topical pharmacologically active treatments remain the first choice in AI-PIH, energy-based devices such as lasers and light sources may be a helpful adjunct to therapy or alternatives in case of topical treatment failure, especially in the darker skin population (53). Laser treatment alone or in combination with topical treatments may provide some responses (42, 54). For AI-PIH treatment in patients with Phototype IV to VI, lasers should be considered in second position after topical agents, due to the variable response, cost, and potential complications. For post-inflammatory hyperpigmentation resistant to topicals, laser devices, particularly neodymium:yttrium-aluminum-garnet and fractional photothermolysis systems, may provide treatment in patients with Phototype IV to VI using suitable parameters (55). However, currently there is little evidence in the literature concerning the benefit of light and laser devices in AI-PIH in all skin types (56). Q-switched nano- and pico-seconds lasers, intense pulsed lights that can be used with low fluences, should be favoured, while ablative lasers should be avoided. Green (510 nm, 532 nm), red (694 nm), or near-infrared (755 nm, 1,064 nm) lasers are pigment-specific and generate light used to selectively target intracellular melanosomes (57). The absorption spectrum of melanin lies between 250 nm and 1,200 nm. With laser energy pointing at deeper targets, it is absorbed within the pigmented epidermis. As a side effect, dyschromia, blistering, and scars may be observed, principally in patients with darker skin (58).

Superficial peelings using salicylic and glycolic acid alone or in combination may be useful to reduce AI-PIH marks (59, 60). Such peelings may be used alone or in combination with lasers.

Currently, various anti-pigmentation agents are formulated in dermocosmetics (39, 46, 61–63). Dermocosmetics containing anti-pigmentation agents as listed in Table III may be proposed in mild forms of AI-PIH as adjuvants to pharmacological or laser treatments, or in combination with peelings (59, 64–67).

Daily skin care should include the use of a mild cleanser with a pH of 5 that is close to the natural pH of the skin in the morning before the use of moisturisers and sun screen, and in the evening prior to applying the acne/AI-PIH treatment (68–70). Furthermore, specific corrective make-up may help to cover AI-PIH marks; however, powder makeup, which may be comedogenic, should be avoided, especially on acne lesions (71).

AI-PIH is worsened by ultraviolet radiation, visible light, and air pollution (72). Therefore, and as explained earlier for preventive measures, concomitant daily use of suitable sunscreens is highly indicated; sun exposure and prolonged exposure to polluted air should also be avoided (40).

**Fig. 2:**
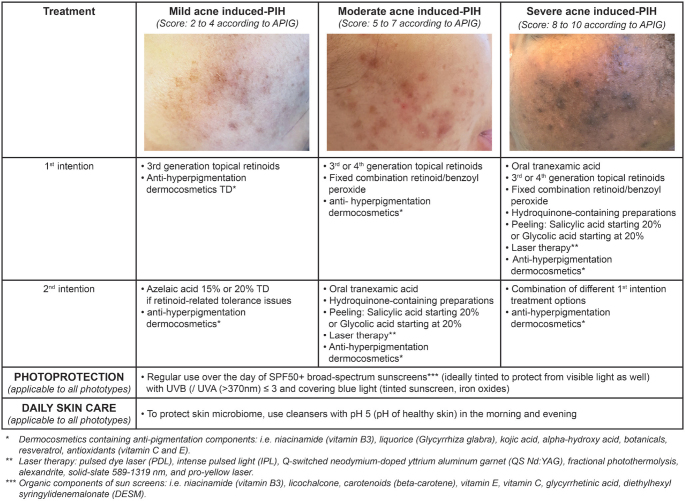
Treatment algorithm Acne PIgmentation Grading.

Despite existing treatment options, no treatment algorithm to treat AI-PIH according to phototype currently exists. We herewith propose an easy-to-follow AI-PIH treatment algorithm (**Fig. 2**). This algorithm is based on both the literature and our clinical practice as experts in acne and associated skin conditions, including AI-PIH. Moreover, it is based on the APIG severity grading, as well as currently available acne treatments, especially retinoids alone or in fixed combinations having shown anti-AI-PIH activities combined with quick efficacy onset in inflammatory lesions and thus preventing AI-PIH, as well as hydroquinone-containing formulations (where available to treat hyperpigmentation), peelings, and different dermocosmetics (35). As for currently proposed acne treatment algorithms we differentiated between mild, moderate, and severe AI-PIH, based on the APIG scoring tool and requiring different treatment approaches. These treatment approaches were categorized as first- or second-intention treatments, completed by photoprotective sunscreens with filters covering UVA1 as well as blue light and daily skin care. The level of treatment evidence is based on currently available data and on our experience.

### Education

Patient education is an important issue to consider in the management of AI-PIH.

With AI-PIH affecting all ages and all phototypes, a global approach to limit AI-PIH is necessary in order to persuade patients to adhere to the preventive and curative treatments of their AI-PIH as much as possible (73).

Therefore, and prior to any therapeutic approach, it is important to discuss the patients’ reason for attending their consultation and the risks for not considering AI-PIH. Especially in young patients, a proactive patient-made decision may be more supportive in managing AI-PIH than a “parent-driven” visit. In the latter situation, the dermatologist may have to primarily create awareness and then educate the patient on the permanent consequences of untreated or inadequately treated acne.

## DISCUSSION

Together with scars, AI-PIH is certainly the most frequently-observed physical consequence of acne. While scars may be observed in all phototypes, AI-PIH is more frequently observed in patients with Phototype IV or above (43). Despite its frequency, the physiopathology of AI-PIH still remains insufficiently investigated and its management as a skin condition has still not deserved adequate attention (16). Probably due to this, to date, no proper AI-PIH grading tool or treatment algorithm has been developed for daily clinical practice, rendering the choice of the most suitable treatment a subjective choice of the dermatologist and the patients, potentially resulting in an insufficient treatment outcome or side effects.

Currently only 1 grading tool exists to assess AI-PIH, the Post-Acne Hyperpigmentation Index (PAHPI) (35). However, this tool is mainly used for clinical study purposes and has not been designed for daily clinical practice. For this reason, and to fill this gap, we have developed the APIG (Acne Pigmentation Grading) tool, which, based on easily obtainable information, allows AI-PIH severity to be quickly and simply graded during the patient’s consultation. This grading considers both the most important factors related to AI-PIH and its intensity.

Moreover, we have provided AI-PIH prevention means and an AI-PIH treatment algorithm, based on the APIG tool. Prevention of AI-PIH is a major issue, especially in patients with Phototypes IV to VI. The 2 most important means that help to prevent AI-PIH include fast treatment onset for inflammatory lesions and avoiding irritation caused by the treatment. Therefore, topical treatment should only be applied in the evening, avoiding firm rubbing or massage. In the morning, patients should apply an adapted moisturiser cream. The use of a cleanser with a pH close to that of the skin (around 5) completes the daily care. To treat AI-PIH, the proposed algorithm has been based on currently used anti-pigmentation agents, peelings, and laser procedures. All have been described to be beneficial in the management of AI-PIH, but without having been categorized according to their anti-pigmentation strength.

Not surprisingly, retinoids are the cornerstone of AI-PIH management (39, 44–47). However, other agents exist to treat AI-PI, such as AA, oral tranexamic acid and HQ, the gold standard, and various agents which are formulated as dermocosmetics (41, 61, 74–76). All of them have their place in the management of AI-PIH.

Of course, APIG, as well as the prevention and treat-ment algorithm, may have to be adjusted during a following phase according to experiences encountered, and with new treatment options becoming available. However, we believe that this first phase will greatly support the dermatologist in making the best treatment proposition for their patients suffering from AI-PIH.

## Data Availability

The data that support the findings of this study are available from the corresponding author upon reasonable request.
